# Severe Acute Respiratory Syndrome Coronavirus 2 (SARS-CoV-2) antigen detection in the Emergency Department: data from a pediatric cohort during the fourth COVID-19 wave in Italy

**DOI:** 10.1186/s13052-022-01343-1

**Published:** 2022-08-26

**Authors:** Angela Pepe, Francesco Valitutti, Deborah Veneruso, Martina Bove, Anna Giulia Elena De Anseris, Lucia Nazzaro, Pasquale Pisano, Daniela Melis, Claudia Mandato

**Affiliations:** 1 Department of Medicine, Surgery and Dentistry “Scuola Medica Salernitana”, Pediatrics Section, University of Salerno, Baronissi (Salerno), Italy; 2 Pediatric Unit, University Hospital “San Giovanni di Dio e Ruggi d’Aragona”, Salerno, Italy; 3 EBRIS (European Biomedical Research Institute of Salerno), Salerno, Italy

**Keywords:** SARS-CoV-2, Rapid antigen diagnostic tests (RADTs), Children, Emergency department

## Abstract

**Background:**

Severe Acute Respiratory Syndrome Coronavirus 2 (SARS-CoV-2) pandemic has been challenging health care systems and made it necessary to use rapid and cost-effective testing methods, particularly in Emergency Department (ED) settings. Rapid Antigen Diagnostic Tests (RADTs) are a valid alternative to the gold standard RT-PCR, even in pediatric populations. This retrospective observational study has been conducted on a pediatric cohort afferent to the ED of the San Giovanni di Dio and Ruggi d’Aragona University Hospital in Salerno, tested at Point of Care with RADT Panbio® (Abbott), from September 1^st^, 2021 to February 28^th^, 2022, analyzing the positivity rate and clinical features of the cohort, also in reference to the rise of positive cases observed in the aforementioned period, and to the introduction in Italy of SARS-CoV-2 vaccination for children and teens on December 16^th^, 2021.

**Methods:**

Data regarding access to the pediatric ED were extracted from the hospital’s electronic database system. Parallel to this, we conducted a narrative literature search using PubMed database focusing on the use of RADT in pediatric ED and compared our data with the national pandemic trend.

**Results:**

During the observation period, 1890 patients were tested for the presence of SARS-CoV-2 with RADT and the 2.7% of children resulted positive, with a peak in January 2022. The main symptoms in positive patients were: fever (*n* = 34; 66.7%), cough (*n* = 11; 21.5%), headache (*n* = 4; 7.8%), chest pain (*n* = 2; 3.9%) and abdominal pain (*n* = 1; 2%). Patients were divided into three different age groups (A, B, C) basing on the different access timing to vaccination; no statistically significant difference was detected in the distribution of positivity in these three groups (*p* > 0.05). Number of positive children in group A was greater in the post-vaccine group. Our data are concordant with the national trend of the pandemic showing a fourth wave peak in January 2022.

**Conclusion:**

The use of RADT as a first point-of-care screening may be helpful, time-saving and cost-sparing. Our study shows that, during the observation period, most children admitted to the ED for fever, actually tested positive for SARS-CoV-2 with a statistically greater difference than negative children. Instead, number of patients admitted for cough was statistically higher among negative than positive ones, probably due to the circulation of other respiratory viruses in children.

## Introduction

Severe Acute Respiratory Syndrome Coronavirus 2 (SARS-CoV-2) pandemic has been challenging health care systems worldwide in multiple ways, one of which has been the increasing necessity of rapid, cost-effective and largely available testing methods, particularly in Emergency Department (ED) settings [1]. It is widely known that Reverse Transcription-Polymerase Chain Reaction (RT-PCR) performed on nasopharyngeal samples is the gold standard to detect the presence of SARS-CoV-2 infection [2].

Rapid Antigen Diagnostic Tests (RADTs), however, have been shown to be a valid alternative to RT-PCR, even in pediatric populations, proving adequate sensibility and specificity [3, 4]. Furthermore, RADT immunoassay’s sensibility increases when conducted on symptomatic children, up to a 80–90% rate [5, 6].

Among the pediatric population, SARS-CoV-2 infection appears to be milder. In comparison to adults, in fact, asymptomatic cases are more frequent and hospitalization and mortality rates are substantially lower. COronaVIrus Disease-19 (COVID-19) symptoms in children are mainly aspecific and often overlap with symptoms of other seasonal respiratory viruses, consisting in fever, upper respiratory tract’s mucositis, fatigue, migraine, dry cough, muscle pain, abdominal pain, diarrhea, nausea and vomit. Moderate cases of pneumonia are rare, even though radiological features suggesting pneumonia were found in asymptomatic patients too [7]. Nevertheless, the spread of new viral variants and the rise of vaccination rates represent new possible variables that can influence COVID-19 presentation in children, so further research is needed in this field.

Taking that into account, this retrospective observational study has been conducted on a pediatric cohort afferent to the ED of the San Giovanni di Dio and Ruggi d’Aragona University Hospital in Salerno, tested at Point of Care with RADT Panbio® (Abbott), from September 1^st^, 2021 to February 28^th^, 2022. This paper aims to analyze the positivity rate and clinical features of this pediatric cohort, considering the rise of positive cases observed in the aforementioned period, and the introduction in Italy of COVID-19 vaccination for children and teens on December 16^th^, 2021.

## Methods

### Study population

**Fig. 1:**
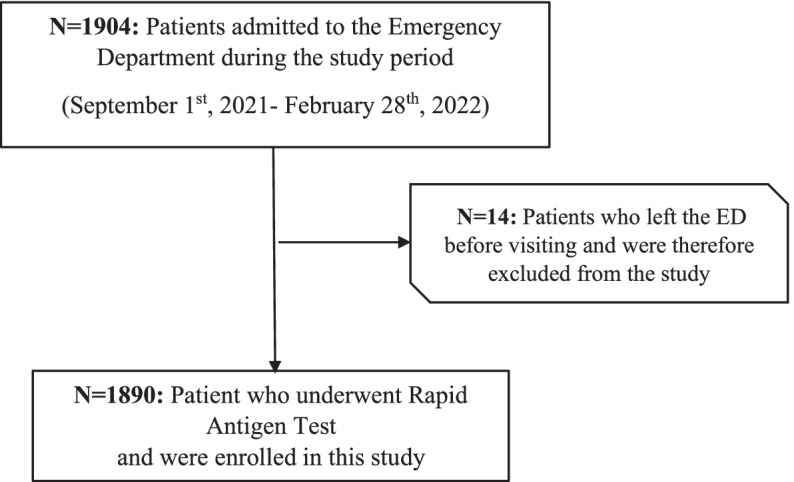
Flow chart of patients enrolled in this study. During the observation period, 1904 pediatric patients were admitted to the Emergency Department (ED) of the San Giovanni di Dio and Ruggi d’Aragona University Hospital in Salerno and 1890 were tested for the presence of SARS-CoV-2 with Rapid Antigen Test. Fourteen patients did not perform the test because left the ED before visiting and were therefore excluded from the study

This single-center retrospective observational study included pediatric patients admitted to the ED of San Giovanni di Dio and Ruggi d’Aragona University Hospital in Salerno between September 1^st^, 2021 and February 28^th^, 2022. Figure 1 contains the flow chart of patients enrolled in this study.

### Procedures

As per our institution protocol, all pediatric patients referred to the ED underwent rapid antigen swab (Panbio®, Abbott) before visiting, as screening method for SARS-CoV-2 infection.

Data were obtained through the hospital’s electronic database system. We extracted demographic, clinical symptoms, management and outcome information from each patient’s electronic medical records. The patients’ vaccinations status was unknown unless clearly reported in the history section.

The results of RT-PCR tests performed, as per protocol, only in hospitalized patients, were also examined. In addition, we conducted a narrative literary review about the use of RADT in pediatric ED using PubMed database (key-words: pediatric, children, emergency department, antigen, COVID-19, SARS-CoV-2) and compared our data with the national pandemic trend extrapolated from GIMBE Foundation monitoring page of the COVID-19 epidemic in Italy [8].

### Ethics

This study was approved by the local ethics committee (“*Comitato Etico Campania Sud*”, regional IRB protocol N. 73 approved on 20/05/2020 meeting for retrospective analysis on ED visits for children); informed consent was not obtained due to the retrospective and anonymised approach which did not change the clinical practice nor provoked any privacy leak for confidential data. The research was conducted according to the Declaration of Helsinki regarding the Ethical Principles for Medical Research Involving Human Subjects.

### Statistical analysis

Statistical analysis was performed using Excel and GraphPad. Data are presented as absolute numbers, percentage and means. Chi-squared tests were used to compare subgroup data as appropriate. A *p* < 0.05 was considered significant for all the tests performed.

## Results

During the observation period (September 1^st^, 2021 - February 28^th^, 2022), 1904 pediatric patients (age 0–14 years) were admitted to the ED of the San Giovanni di Dio and Ruggi d’Aragona University Hospital in Salerno and 1890 (1040 males) were tested for the presence of SARS-CoV-2 with RADT (14 patients did not perform the test because left the ED before visiting and were therefore excluded from the study). The total cost for antigen swabs was about 2500 euros.

The 2.7% of children (*n* = 51, median age 43 months, 28 males) tested positive, with a peak in January 2022. Hospitalization was required in 6 positive children (11.8%; median age 19 months) and 376 negative ones (20.4%; median age 43.5 months): the 6 patients tested positive for RADT were confirmed positive for the RT-PCR test; however, among the 376 negative ones, 3 patients were actually positive for the RT-PCR test (false negative). The specificity of RAD testing was 100% and the sensitivity was 94% (3 false negative patients, 51 true positives ones). Main characteristics of the study cohort are summarized in Table 1.

**Table 1:** Demographic and clinical characteristics of patients admitted to ED and included in the study

	Total	Positive	Negative	***P value***
Patients (%)	1890	2.7% (*n* = 51)	97.3% (*n* = 1839)	
**Demographic data**				
Median Age (months)	89	43	42.5	
Range Age (years)	0–14	0–14	0–14	
Male (%)	55% (*n* = 1040)	55% (*n* = 28)	55% (*n* = 1012)	> 0.05
Female (%)	45% (*n* = 850)	45% (*n* = 23)	45% (*n* = 827)	> 0.05
**Clinical features**				
Fever (%)	43.9% (*n* = 830)	66.7% (*n* = 34)	43.3% (*n* = 796)	0.0009
Cough (%)	35.7% (*n* = 675)	21.5% (*n* = 11)	36.1% (*n* = 664)	0.032
Headache (%)	2.5% (*n* = 48)	7.8% (*n* = 4)	2.4% (*n* = 44)	0.0147
Chest pain (%)	2.0% (*n* = 39)	3.9% (*n* = 2)	2.0% (*n* = 37)	> 0.05
Abdominal pain (%)	3.0% (*n* = 57)	2% (*n* = 1)	3.0% (*n* = 56)	> 0.05
Other symptoms (%)		27.4% (*n* = 14)		
Vaccination group A (%)	72% (*n* = 1366)	70.5% (*n* = 36)	72.3% (*n* = 1330)	> 0.05
Vaccination group B (%)	22% (*n* = 409)	25.5% (*n* = 13)	21.5% (*n* = 396)	> 0.05
Vaccination group C (%)	6% (*n* = 115)	4% (*n* = 2)	6.2% (*n* = 113)	> 0.05
Hospitalized	20.2% (*n* = 382)	11.8% (*n* = 6)	20.4% (*n* = 376)	> 0.05
Accessed to the ED by ambulance	2.7% (*n* = 51)	25.5% (*n* = 13)	2% (*n* = 38)	< 0.0001

The main symptoms in positive patients were: fever (*n* = 34; 66.7%), cough (*n* = 11; 21.5%), headache (*n* = 4; 7.8%), chest pain (*n* = 2; 3.9%) and abdominal pain (*n* = 1; 2%). Seven patients reported respiratory distress among the presenting symptoms; however, only in one child it was actually confirmed by physical examination (tachypnea, nasal flaring, use of accessory muscles).

Fourteen positive patients (27.4%) were admitted to the ED in January for other medical problems (seizures, lipothymia, accidental ingestion of toxic substances, etc).

Patients were divided into three different age groups basing on the different access timing to vaccination:

*- Group A*, children under 5 years: this group included 1366 patients, 36 positive (70.5%; 22 males) and 1330 negative (72.3%; 728 males) ones. This group was exempt from the vaccination campaign.
*- Group B,* children ages 5 to 11: this group was composed by 409 patients including 13 positive (25.5%; 6 males) and 396 negative children (21.5%; 220 males). These patients in Italy accessed the vaccination campaign on December 16^th^, 2021.
*- Group C*, children ages 12 to 14: this group included 115 patients, 2 positive (4%; 2 males) and 113 negative ones (6.1%; 64 males). These children had the opportunity to receive the SARS-CoV-2 vaccine from June 2021.

**Fig. 2:**
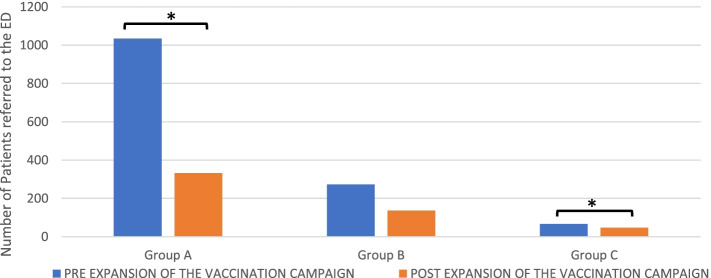
Number of accesses to the Emergency Department (ED). The significant difference (*p* < 0.01) in the number of accesses pre / post expansion of the vaccination campaign in groups A and C is demonstrated with *

**Fig. 3:**
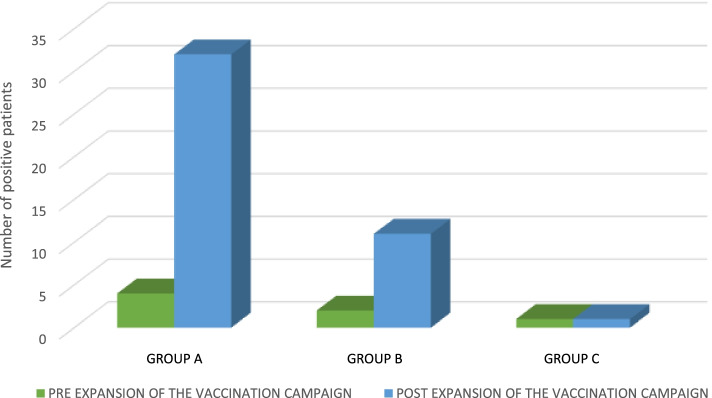
Distribution of positive patients in groups **A**, **B**, **C**. Differences between groups were not significant (*p* > 0.05)

**Fig. 4:**
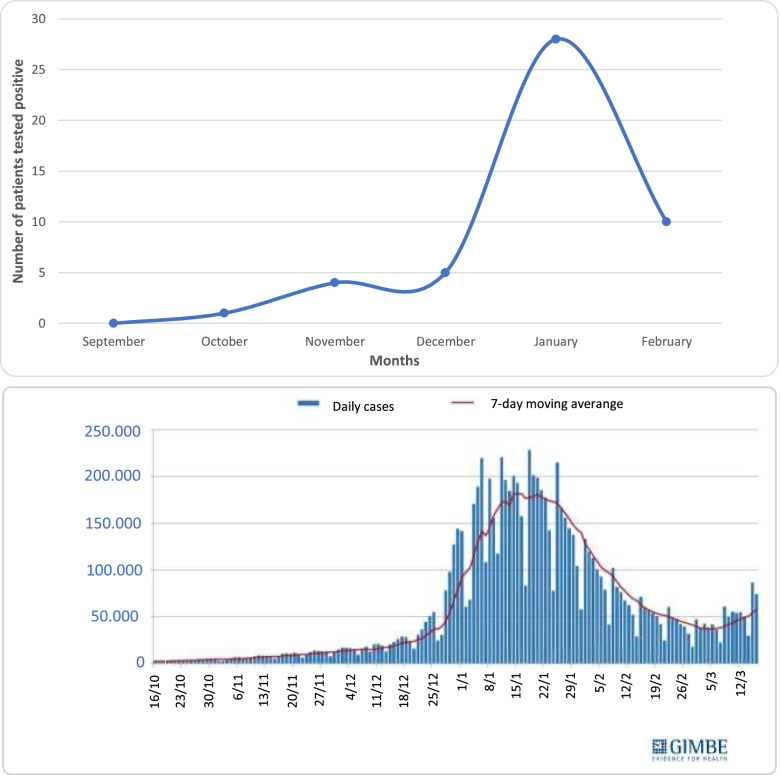
Cases distribution in the observation period (top figure) and Italian trend of the pandemic (bottom figure). As shown in the figures, the peak of positive cases in both our cohort and national trend occurred in January 2022

**Fig. 5:**
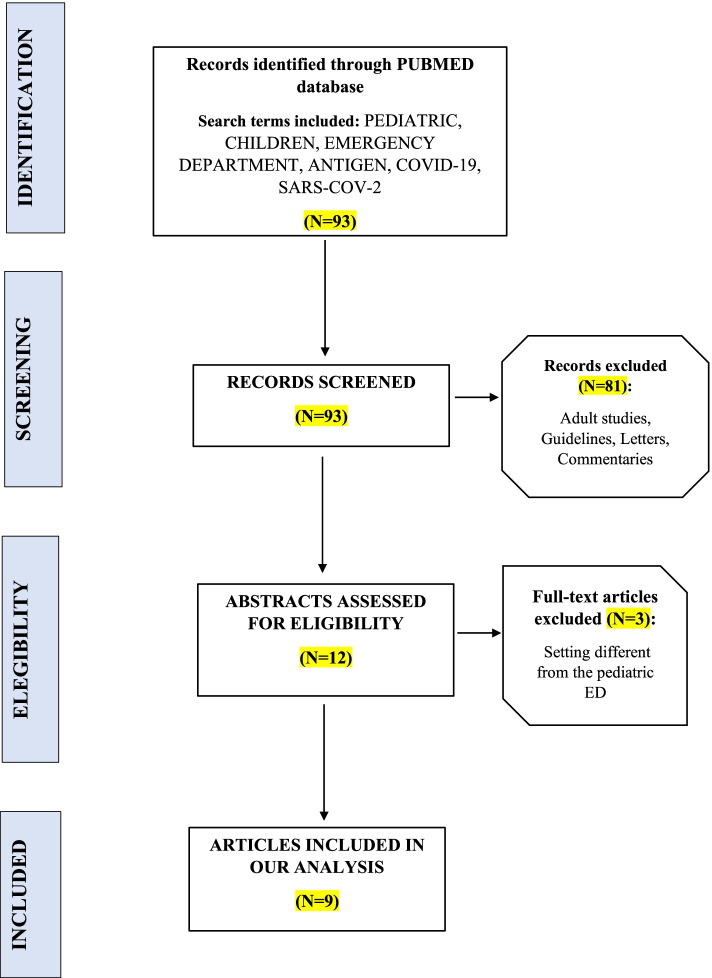
Flow diagram regarding study selection

The distribution of positivity in these three groups showed no statistically significant differences (*p* > 0.05). Related to the expansion of the nation’s vaccination campaign to children as young as 6–11 (16^th^ December 2021), we observed in groups A and C a number of accesses pre-expansion of vaccination campaign significantly greater (*p* < 0.01) (Fig. 2) and a non significant (*p* > 0.05) difference in the number of positives pre / post expansion of the vaccination campaign (Fig. 3). The number of positive patients in group A was greater in the post-vaccine group. Our data are concordant with the national trend of the pandemic showing a fourth wave peak in January 2022 (Fig. 4) [8]. Literature research on this topic was carried out on PubMed database using the following key words: pediatric, children, emergency department, antigen, COVID-19, SARS-CoV-2 (Fig. 5). A total of 93 articles was retrieved from PubMed database; 84 were excluded as they were guidelines, adult studies (> 18 years), letters, commentaries, studies concerning the use of RADTs in a setting other than ED. A final set of 9 articles (2 retrospectives and 7 prospectives) was suitable for the scope of our review as they described the use of RADTs in a pediatric emergency department setting (Table 2). These papers compared the performance of RADTs with PCR test as the gold standard. As shown in the table, RADTs showed a wide range of sensitivity (45.4–94.1%) though all of them presented a good specificity (range 91.9–100%).

**Table 2:** Literature review regarding the use of RADTs in the pediatric ED

Authors	Type of study	Type of RADT	Number of Patients	Sensitivity (%)	Specificity (%)
**Denina et al.** [9]	retrospective	LumiraDx TM Platform	191	94.1	91.9%
**Ollier et. al.** [6]	prospective	Biospeedia antigen test	1009	69.9%	99.9%
**Jung et al.** [10]	prospective	Biosynex COVID-19 Ag BSS	308	87.9%	98.5%
**Carbonell -Sahuquillo et al.** [3]	prospective	PanbioTM COVID-19 Ag Rapid Test Device	357	70.5%	100%
**Villaverde et al.** [5]	retrospective, multicenter	Panbio COVID-19 Ag Rapid Test Device (Abbott Rapid Diagnostic)	1620	45.4%	99.8%
**Reichert et al.** [4]	prospective	SD BioSensor Inc., Suwon, South Korea	710	75%	99.4%
**González-Donapetry et al.** [11]	prospective	Abbott Rapid Diagnostics Jena GmbH, Jena, Germany	440	77.78%	100%
**Lanari et al.** [12]	prospective (using 2 types of RAD kit)	COVID-19 Ag Fia Kit AFIAS COVID-19 Ag kit	386 792	53.8% 86.4%	99.7% 98.3%
**Mockel et al.** [13]	prospective	Roche SARS-CoV-2 rapid antigen test, Penzberg, Germany	202	72%	99.4%

## Discussion

From 2019, SARS-CoV-2 was recognized as the etiological agent of COVID-19 [14]**,** making necessary rapid detection of infected patients.

PCR- RT is the gold standard for diagnosing SARS-CoV-2 infection. However, especially in the ED, it is important the use of rapid screening methods to limit viral transmission and prevent COVID-19 spreading in hospital settings [15].

RADTs represent an efficient alternative as a first-line diagnostic method combining both advantages: low price and quick results. As the detected antigens are expressed during viral active replication, RADT accuracy is closely linked to the viral load and it can be used to identify acute infection [16].

The guidance released on 11^th^ September by the World Health Organization (WHO) [17] recommended use of RADTs for diagnosis of COVID-19 when the RT-PCR test is not available underlying that it must have a sensitivity of at least 80%.

Many studies are available in literature about the use of RADTs in the pediatric ED, mainly focused on comparison with the gold standard RT-PCR.

Carbonell- Sahuquillo et al. [3] evaluated the Panbio™ COVID-19 Ag Rapid Test Device as a point-of-care diagnostic tool for COVID-19 at a pediatric ED. The authors underline that, even if the assay did not meet one of the criteria (at least 80% sensitivity) recommended by WHO interim guidance for RADT diagnosis of SARS-CoV-2 infection, its performance in identifying children with high SARS-CoV-2 RiboNucleic Acid loads in the upper respiratory tract, which associate with contagiousness, makes it a valuable tool for the management of children with suspected COVID-19 at the ED.

Ollier et al. [6] found that sensitivity of RADTs increased from 69.6 to 82.9% when performed on symptomatic children suggesting that RADTs sensitivity depends on the entity of nasal viral load at testing time, which is higher in the first few days of infection when patients are most likely to be symptomatic. Their results are in line with what has been observed by Mockel et al. [13]: false negatives obtained with RADTs coincide with low viral load confirmed by RT-PCR.

The Panbio™ rapid antigen test kit for SARS-CoV-2 (Abbott Diagnostic GmbH, Jena, Germany) is a qualitative test using specimens from nasopharyngeal swabs. The manufacturer declared in patients with symptoms a sensitivity of 93.3% overall and 98.2% in those RT-PCR cycle threshold ≤33, and a specificity of 99.4% [18]. These data agree with the findings in our cohort of hospitalized, and therefore symptomatic patients: the Panbio™ rapid antigen test showed a sensitivity of 94% and a specificity of 100%.

Assuming that RADTs reach high sensitivity mainly when patient is contagious, and that in EDs screening for SARS-CoV-2 is aimed precisely at avoiding the viral transmission during visits and procedures that the patient must carry out quickly making it difficult to obtain the result of a test analyzed by RT-PCR, our study wants to provide a description of the epidemiological picture during the “fourth wave” of the Pandemic evaluating the performance of the Panbio™ COVID-19 RADT for detecting SARS-CoV-2 and describe the cohort’s clinical features, considering positive cases observed in the aforementioned period. Moreover, our efforts aim at evaluating the impact of the introduction in Italy of COVID-19 vaccination for children and teens seen at ED during the study period. Regarding the demographic characteristics, we observed an overlapping median age between positive and negative ones. As regards clinical features, the number of patients admitted to the ED for fever and for headache was statistically higher in those who tested positive than negative counterpart (*p* < 0.05). On the other hand, the number of patients who came to ED for cough was statistically higher in negative patients than in the positive ones. This unexpected finding can be explained by public health measures to tackle Coronavirus spread, such as the obligation of face masks, which did not involve children. We can speculate that this may be attributable to contemporary circulation of other respiratory viruses.

No significant difference was found regarding hospitalization rates between positive and negative groups, but we can trace back this data to the scarcity of pneumonia related to COVID-19 in pediatric patients, since the disease in children runs mildly in the majority of cases. According to triage severity, we did not find a statistically significant difference in patients admitted between positive and negative. On the other hand, the number of positive patients conducted to the ED by ambulance was significantly greater compared to the negative group; in about 70% of cases the reason for accessing ED was fever. This aspect may reflect parents’ fear of having contracted the virus and of having to manage this condition at home in a time when accessing to territorial pediatric care was more difficult and subject to the execution of a COVID swab. Moreover, our subcohorts (A, B, C) according to the different timing to vaccination allowed us to note that the distribution of positivity is not statistically significant in the three groups (*p* > 0.05).

It is interesting to note that, in the period before vaccine implementation among children, the number of accesses to the ED was significantly higher for groups A and C than in the subsequent period, probably due to the pandemic curve with a peak of the “fourth wave” in January 2022. However, group B did not show this trend. Furthermore, epidemiological context must be considered: by the start of the study period, the 7-day Italian incidence rate was 54 per 100,000 on September 6–12, 2021 and the peak of the fourth wave was reached in the week 10–16 January 2022 with an incidence rate of 2021 per 100,000. At the end of the observational period a 7-day incidence rate of 433 per 100,000 was recorded in Italy [8].

According to our knowledge, this is the first study describing the clinical characteristics of a pediatric cohort afferent to the ED who tested positive for a rapid antigen test also referring to the progress of the vaccination campaign in children and the national trend of the pandemic.

It is not possible to express an absolute opinion about the vaccination campaign’s impact because the effects are to be sought after the observation period of this study.

Our study has some limitations: first, no data about time of onset of symptoms have been collected and all patients were subjected to RADT indifferently; furthermore, the most important limitations of this study are its retrospective design and the lack of RT-PCR data for all patients because this test, as per our protocol, is performed only in children requiring hospitalization. However, we have tried to limit the effects of these by opting to adopt a descriptive rather than an analytical approach, and by making within-group comparisons.

## Conclusions

In conclusion, the use of rapid antigen test as a first point-of-care screening, later integrated with molecular test, may be helpful, time-saving and cost-sparing, and could reduce the time children stay in the waiting room and reducing the hospital infection with SARS-CoV-2 or other pathogens. Clearly it must be designed in synergy with other containment measures, such as the use of masks and frequent ventilation of the rooms. Our data suggest that, during the observation period, most children admitted to the ED for fever actually tested positive for SARS-CoV-2 with a statistically greater difference than negative children.

Instead, number of patients admitted for cough was statistically higher among negative than positive ones, probably due to the circulation of other respiratory viruses in children. In fact, the highest number of positivity was observed in children under the age of 5 who had not been involved in the vaccination campaign and were not required to wear personal protection. Learning also from this experience and the diagnostic yield of RADTs, we encourage health care systems to move toward more integrated models between hospitals and community services, perhaps with safer rapid swab hubs benefitting both ED and primary care.

## Data Availability

All data generated or analyzed during this study are included in this published article.

## Abbreviations

SARS-CoV-2: Severe Acute Respiratory Syndrome Coronavirus 2
ED: Emergency Department
RADTs: Rapid Antigen Diagnostic Tests
RT-PCR: Reverse Transcription-Polymerase Chain Reaction
COVID-19: COronaVIrus Disease-19
WHO: World Health Organization
